# Exploring the Association Between Paralytic Ileus and Endoscopic Retrograde Cholangiopancreatography Complications Using the National Inpatient Sample Database

**DOI:** 10.7759/cureus.30319

**Published:** 2022-10-15

**Authors:** Vincent Wong, Reza Hashemipour, Anjella Manoharan, Sushil Ahlawat

**Affiliations:** 1 Internal Medicine-Pediatrics, Rutgers University New Jersey Medical School, Newark, USA; 2 Gastroenterology and Hepatology, Rutgers University New Jersey Medical School, Newark, USA; 3 Gastroenterology and Hepatology, Rutgers University, Newark, USA

**Keywords:** post-procedure complication, gastrointestinal ileus, interventional endoscopy, endoscopic retrograde cholangiopancreatography (ercp), paralytic ileus

## Abstract

Introduction

Paralytic ileus (PI) is often seen in critically ill hospitalized patients. Those with pancreaticobiliary diseases will require endoscopic retrograde cholangiopancreatography (ERCP) for management. Here, we will explore the association between patients with paralytic ileus who underwent ERCP and post-procedural complications, which has not been done before.

Methods

Patients who underwent ERCP between 2007 and 2017 in the National Inpatient Sample database were selected. Cases were matched 1:1 by age, gender, race, and the Elixhauser comorbidity index for patients with and without pre-procedural paralytic ileus. Primary outcomes were associations between paralytic ileus and length of stay, payor status, and average total charges. Secondary outcomes were associations between paralytic ileus and post-ERCP complications (infection, pancreatitis, cholangitis, cholecystitis, perforation, hemorrhage), and overall mortality. The Chi-squared analysis was used to compare categorical data, and the independent t-test was used for continuous data. Regression analysis was used to assess primary and secondary outcomes.

Results

Of 2,008,217 hospitalized patients from 2007 to 2017, 43,643 patients had paralytic ileus and 43,859 patients did not, before undergoing ERCP. There were no differences in age, gender, race, or the Elixhauser comorbidity index. The differences in the length of stay, payor status, and total charges were significant (p<0.001). Patients with paralytic ileus had increased risks of post-ERCP infection, pancreatitis, cholangitis, cholecystitis, perforation, hemorrhage, and overall mortality (p<0.001).

Conclusions

Patients hospitalized with paralytic ileus who underwent ERCP had a longer length of stay, higher total charges, and were less compensable. They also had increased risks for post-ERCP infection, pancreatitis, cholangitis, cholecystitis, perforation, hemorrhage, and overall mortality, which can be from critical illness and the systemic inflammatory response.

## Introduction

Paralytic ileus (PI) is common in critically ill patients after surgery due to an inflammatory response, electrolyte abnormalities, or medications [1-3]. It can lead to high morbidity and mortality if not acutely cared for, diagnosed early, and managed appropriately [1,4-6]. Pancreaticobiliary diseases can also cause patients to become severely ill and ERCP has been the mainstay for treatment [6-10]. By entering the biliary tree and using contrast dye, ERCP can better visualize obstructions, strictures, and structural anomalies that may be difficult to assess with other imaging modalities [5-8,11]. However, in 6-10% of patients, this technique can lead to complications such as infection, perforation, cholangitis, pancreatitis, and bleeding [10,12]. Since this has not yet been described in the literature, we hypothesize that the pathophysiological changes seen in patients with paralytic ileus may cause more post-procedural complications. Here, we will use the National Inpatient Survey (NIS) Database to explore the association between patients with paralytic ileus who underwent ERCP and post-procedural complications.

## Materials and methods

Data source

The NIS Database is a publicly available database from the Agency for Healthcare Research and Quality (AHRQ) documenting de-identified patient information from inpatient hospitalizations in the United States. No institutional review board approval was needed for this retrospective study because of publicly available information.

Study population and design

The NIS database was used to look at patients hospitalized between 2007 and 2017. Patients over 18 years old who underwent ERCP were selected using their respective International Classification of Diseases (ICD) procedure codes (Appendix - Table 4). ICD-9 was used for data from 2007 to 2015, and ICD-10 was used for data from 2015 to 2017. Patients were grouped into those who had the diagnosis of paralytic ileus using their ICD-9 and ICD-10 diagnosis codes (Appendix - Table 5), and those who did not. Patients with post-procedural complications were identified using their respective ICD-9 and ICD-10 diagnosis codes simultaneously with the ERCP diagnosis codes (Appendix - Table 5). Our dataset was weighted and cases were matched 1:1 by age, gender, race, and Elixhauser comorbidity index before statistical analyses were done.

Demographic information on the patients’ age, gender, race, and Elixhauser comorbidity index was collected. Patients were grouped into the following categories by age: 18-27 years old, 28-37 years old, 38-47 years old, 48-57 years old, 58-67 years old, 68-77 years old, 78-87 years old, and those older than 88 years old. The Elixhauser comorbidity index was categorized into those with a mortality score of ≤−1, 0, 1-5, 6-10, and ≥11. Data on length of stay, payor status, and average total charges were also collected and analyzed. The Chi-squared analysis was used to determine the statistical significance between categorical data sets, and the t-test was used to determine the significance of continuous data.

Multivariate logistic regression analysis (adjusted for age, gender, race, and the Elixhauser comorbidity index) was performed to determine the odds ratio, significance, and 95% confidence intervals for post-ERCP infection, pancreatitis, cholangitis, cholecystitis, perforation, hemorrhage, and overall mortality. Univariate logistic regression analysis was used to determine independent predictors of post-ERCP complications (infection, pancreatitis, cholangitis, cholecystitis, perforation, and hemorrhage) in patients with pre-procedural paralytic ileus. Because of the nature of the NIS database, complication diagnoses were selected by removing the first two sets of diagnosis codes. This filtered out patients who had these as their primary diagnoses for hospital admission. The method used has been published by Choi et al. and Solanki et al. [9,13].

Outcomes

The primary goals of this study were to determine the associations between paralytic ileus with the length of stay, payor status, and average total charges. The secondary outcomes were to determine the associations between patients with paralytic ileus and post-ERCP complications (infection, pancreatitis, cholangitis, cholecystitis, perforation, hemorrhage, and overall mortality).

Statistical analysis

Statistical Package for the Social Sciences (IBM Corp., Released 2021; IBM SPSS Statistics for Macintosh, Version 28.0, Armonk, NY) was used for data analysis. Cases were matched 1:1 by age, gender, race, and the Elixhauser comorbidity index. The Chi-squared and independent sample t-tests were used to determine the significance of the patients’ demographic information, length of stay, payor status, and average total charges. Multivariate logistic regression was used to determine the odds ratios (OR), significance, and 95% confidence intervals of post-ERCP complications. Univariate logistic regression was used to calculate the OR, significance, and 95% confidence intervals for independent predictors of post-ERCP complications. Only p-values <0.05 were considered significant. 

## Results

**Figure 1:**
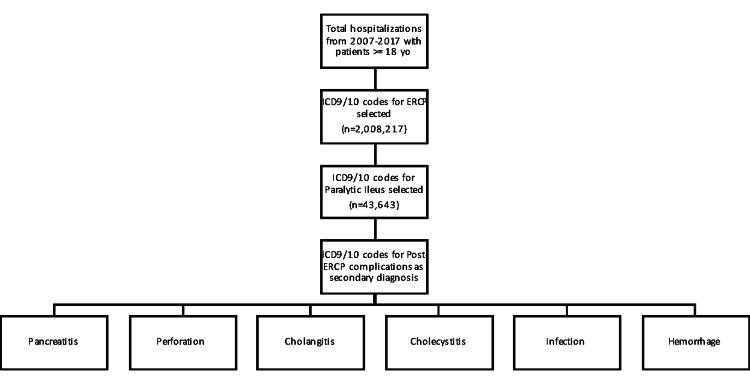
Study design schema for selecting patients with and without paralytic ileus who underwent ERCP and had post-procedural complications

Our sample population was selected from the NIS database by combining de-identified patient information from 2007 to 2017. There was a total of 2,008,217 weighted patient cases over 18 years of age (Figure 1). Patients who underwent ERCP procedures were selected based on their respective ICD-9 and ICD-10 codes. Patients were subdivided into those who had paralytic ileus and those who did not. Patients were matched 1:1 for age, gender, race, and Elixhauser comorbidity index. Those who had overall ERCP complications as secondary diagnoses were selected: post-ERCP infection, pancreatitis, cholangitis, cholecystitis, perforation, and hemorrhage based on their ICD-9 and ICD-10 codes, as well as overall mortality.

There were 43,643 patients in the PI group and 43,859 patients in the ‘control’ group, or those without paralytic ileus. Both groups did not show differences in significance for age, gender, race, and the Elixhauser comorbidity index (Table 1). Most of the patients were between 58-67 (20.2%) and 68-77 (21.8%) years old, had a mean age of 63 years old, were White (72.0%), and had a male-to-female ratio of 52.7% vs. 47.3%, respectively. The average Elixhauser comorbidity index for patients with paralytic ileus was 10.0 and that in the control group was 9.4.

**Table 1:** Demographics of patients with and without paralytic Ileus who underwent endoscopic retrograde cholangiopancreatography NS: not significant

	Control, N=43,859 (%)	Paralytic Ileus, N=43,643 (%)	Significance
Age (years)	Mean = 63.0 (SD=17.5)	Mean = 63.0 (SD=17.4)	NS
18–27	1685 (3.8)	1666 (3.8)	
28–37	2792 (6.4)	2780 (6.4)	
38–47	4354 (9.9)	4312 (9.9)	
48–57	6537 (14.9)	6501 (14.9)	
58–67	8839 (20.2)	8828 (20.2)	
68–77	9569 (21.8)	9499 (21.8)	
78–87	7754 (17.7)	7735 (17.7)	
≥88	2328 (5.3)	2323 (5.3)	
Gender			NS
Male	23,090 (52.6)	22,994 (52.7)	
Female	20,768 (47.4)	20,649 (47.3)	
Race			NS
White	31,600 (72.0)	31,433 (72.0)	
Black	4230 (9.6)	4221 (9.7)	
Hispanic	4678 (10.7)	4662 (10.7)	
Asian or Pacific Islander	1862 (4.2)	1864 (4.3)	
Native American	296 (0.7)	288 (0.7)	
Other	1193 (2.7)	1175 (2.7)	
Elixhauser comorbidity index	Mean = 9.4 (SD=10.4)	Mean = 10.0 (SD=11.1)	NS
≤−1	7951 (18.1)	7903 (18.1)	
0	4841 (11.0)	4818 (11.0)	
1–5	4681 (10.7)	4655 (10.7)	
6–10	7190 (16.4)	7160 (16.4)	
>10	19,196 (43.8)	19,107 (43.8)	
Length of stay (in days)	Mean = 7.3 (SD=8.1)	Mean = 14.2 (SD=14.0)	P<0.001
0–9	35,025 (79.9)	20,051 (45.9)	
10–19	6529 (14.9)	14,975 (34.3)	
20–29	1337 (3.0)	4861 (11.1)	
30–39	457 (1.0)	1806 (4.1)	
40–49	263 (0.6)	795 (1.8)	
≥50	252 (0.6)	1159 (2.7)	
Payor			P<0.001
Medicare	22,804 (52.0)	22,850 (52.5)	
Medicaid	4643 (10.6)	3996 (9.2)	
Private	12,920 (29.5)	13,307 (30.5)	
Self-pay	2056 (4.7)	1869 (4.3)	
No charge	249 (0.6)	289 (0.7)	
Other	1155 (2.6)	1254 (2.9)	
Total charges	$74,208.1	$138,178.0	P<0.001

For our primary outcomes, length of stay, payor status, and total charges showed statistical significance (p<0.001) (Table 1). The length of stay of patients with paralytic ileus was higher than those without, with more patients having at least 10 days of hospitalization in the PI group (34.3% vs. 4.9%). Fewer patients in the PI group than in the control group had some form of payment: ‘Medicare’ (52.5% vs 52.0%), ‘Medicaid’ (9.2% vs 10.6%), ‘Private Insurance’ (30.5% vs 29.5%), and ‘Self-Pay’ (4.3% vs 4.7%), which collectively equalled 96.5% vs 96.8%, respectively. Total charges ($138,178.0 vs. $74,208.1) were higher in patients with paralytic ileus than in those without.

Mortality was highest (3.4%), followed by post-ERCP infection (2.3%), pancreatitis (1.0%), cholangitis (1.2%), cholecystitis (0.1%), perforation (0.4%), and hemorrhage (0.3%) (Table 2). The data were significant (p<0.001) for post-ERCP infection (OR = 10.4), pancreatitis (OR = 5.6), cholangitis (OR = 15.3), cholecystitis (OR = 7.4), perforation (OR = 14.6), hemorrhage (OR = 15.2), and overall mortality (OR = 2.1).

**Table 2:** Multivariate regression analysis of patients with paralytic ileus and post-endoscopic retrograde cholangiopancreatography complications adjusted for age, gender, race, and Elixhauser comorbidity index CI: confidence interval

Type of complication	Number of patients (% total)	Odds ratio (95% CI)	P-value
Infection	1974 (2.3)	10.4 (8.9–12.2)	<0.001
Pancreatitis	859 (1.0)	5.6 (4.6–6.7)	<0.001
Cholangitis	1031 (1.2)	15.3 (11.9–19.6)	<0.001
Cholecystitis	125 (0.1)	7.4 (4.3–12.6)	<0.001
Perforation	310 (0.4)	14.6 (9.3–23.0)	<0.001
Hemorrhage	235 (0.3)	15.2 (9.0–25.8	<0.001
Overall mortality	3015 (3.4)	2.1 (1.9–2.2)	<0.001

In patients with paralytic ileus, independent predictors of ERCP complications were significant for age (OR = 1.01), Elixhauser comorbidity index (OR = 1.03), length of stay (OR = 1.02), had ‘No Charge’ (OR = 2.08), and ‘Other’ (OR = 1.24) payor status (Table 3). Those who were female (OR = 0.78), had ‘Private’ (OR = 0.91) insurance, and were ‘Self-Pay’ (OR = 0.62) were protective factors against post-ERCP complications.

**Table 3:** Univariate regression analysis of patients with paralytic ileus to determine independent predictors of post-endoscopic retrograde cholangiopancreatography complications CI: confidence interval, NS: not significant

	Odds ratio (95% CI)	P-value
Age	1.01 (1.00–1.01)	<0.001
Female	0.78 (0.73–0.84)	<0.001
Race		
White	Reference	
Black	0.86 (0.75–0.98)	0.025
Hispanic	1.17 (1.04–1.31)	0.010
Asian or Pacific Islander	1.50 (1.28–1.76)	<0.001
Native American	0.49 (0.26–0.93)	0.028
Other	0.80 (0.62–1.03)	NS
Elixhauser comorbidity index	1.03 (1.02–1.03)	<0.001
Length of stay	1.02(1.01–1.02)	<0.001
Payor		
Medicare	Reference	
Medicaid	0.99 (0.86–1.12)	NS
Private	0.91 (0.83–0.99)	0.023
Self-pay	0.62 (0.49–0.77)	<0.001
No charge	2.08 (1.49–2.92)	<0.001
Other	1.24 (1.01–1.52)	0.037

## Discussion

Paralytic ileus is a common condition in hospitalized patients with a mortality of up to 6% [1,4]. It is a type of functional ileus characterized by decreased contractions of smooth muscles of the bowel wall [1,3,14,15]. Usually occurring after abdominal or retroperitoneal surgeries, it can also be due to medications (e.g., opioids or neuroleptics), metabolic disorders (e.g., electrolyte disturbances - hypokalemia, diabetes mellitus), or vascular injuries (e.g., hypoperfusion of the bowel) [1-4,16]. Unlike mechanical ileus where there is some form of physical obstruction, paralytic ileus is multifactorial, involving cytokines, neuropeptides, and nitric oxide leading to dysregulation of the sympathetic and parasympathetic control of the gut [1,3,14]. If severe enough, ileus can lead to intestinal obstruction, abdominal distension, and perforation, which is a surgical emergency [1,15]. Treatment mostly involves correcting the underlying causes [1,2,4,14].

Since the 1960s, ERCP has been used for the treatment of choledocholithiasis, acute biliary pancreatitis, cholangitis, pancreatic head masses, and other biliary obstructions using minimally invasive techniques [5,8,11]. However, there have been well-documented complications seen in 6-10% of patients, most commonly pancreatitis (3.5%), perforation (0.6%), cholangitis (1%), infection (0.25%), cholecystitis (0.2-0.5%), hemorrhage (1.3%), and mortality (0.2-0.5%), seen as far as 30 days after the procedure [6,10,12,17,18]. The incidence of complications has increased, which is seen with pancreatitis, from 12.3% in 2007 to 16.5% in 2016, because of the increase in the use of ERCP [5]. There have been studies done on interventions to decrease these rates using guidewire cannulation, stents, and NSAIDs for pancreatitis or antibiotics perioperatively to reduce post-procedural sepsis [5,19]. However, the associations and rates of ERCP complications in patients with existing paralytic ileus have not been described before.

Results from our study show that the overall rate of post-ERCP complications (8.7%) is within the ranges seen by other authors (6-10%) [10,12]. The mortality rate for patients with paralytic ileus who underwent ERCP was 3.4% with an OR of 2.1, which is drastically higher than values reported by Anderson et al. of up to 0.5% [19]. This can be explained by the idea that critically ill patients are more susceptible to developing sepsis, organ dysfunction, metabolic derangements, and cardiorespiratory compromise [1,16,20-24]. Paralytic ileus is independently known to cause sepsis as well, as the decrease in intestinal motility changes the intestinal flora, allowing for the overgrowth of bacteria and gut translocation [22]. Performing ERCP in patients with paralytic ileus can cause gut bacteria to seed the biliary tree, especially if an improperly cleaned endoscope is used [9]. These are likely the causes of the higher number of patients seen with post-ERCP infections (2.3%, OR = 10.4), which can lead to pancreatitis (1.0%, OR = 5.6), cholangitis (1.2%, OR = 15.3), and cholecystitis (0.1%, OR = 7.4), contributing to our higher mortality rate.

Paralytic ileus has also been shown to be associated with an increased risk of post-ERCP perforation (0.4%, OR = 14.6) and hemorrhage (0.3%, OR = 15.2). These results can be explained by a structurally compromised intestinal tract in patients with paralytic ileus. Decreased intestinal motility causes distension, cytokine release, inflammation, and capillary wall leakage, which can lead to gut wall edema or ischemia [22]. These pathophysiological changes can make the duodenum more susceptible to perforation when air or carbon dioxide is injected as the endoscopist uses a side-viewing scope to cannulate the major papilla. Bleeding can occur when cannulation or sphincterotomy is attempted. However, these values are lower than those reported, with 1.3% of patients having post-ERCP hemorrhage and 0.6% having perforation [19]. This is likely due to a lower threshold for the endoscopist to abort the procedure in these high-risk patients.

Other factors to consider in patients with paralytic ileus are the location and timing of ERCP. The literature has shown that ERCP can be done safely at the bedside in the intensive care unit for patients who are on mechanical ventilation and vasopressor support [24,25]. Patients who need procedures done at the bedside require extensive coordination and preparation to move equipment quickly. Since timing can be a priority in an acutely sick patient, the endoscopist may not have all the tools necessary to complete the procedure safely and promptly outside of the gastroenterology suite [24-26]. These can contribute to more complications, subsequently causing the increased lengths of stay and higher total charges seen in our study.

In addition, our data show that increasing age, higher Elixhauser comorbidity index, and longer lengths of stay were independently associated with post-ERCP complications in patients with paralytic ileus. Being female was protective against ERCP complications, which is contrary to some of the literature reporting that being female was a risk factor for post-ERCP pancreatitis [27]. This can be explained by the fact that our population has more males than females, skewing this aspect of our results. Patients with ‘No Charge’ or ‘Other’ forms of payor status were associated with increased complications as well. According to Gabriel et al. and Fowler et al., critically ill uninsured patients were less likely to receive critical care services, have delays in procedures, and therefore have higher rates of mortality [28,29].

The limitations of our study come from the use of the NIS database to identify patients with ERCP complications as secondary diagnoses; the small sample size of patients after switching from ICD-9 to ICD-10 codes in 2015; and the inability to code levels of care during the hospitalization. ICD-9 and ICD-10 do not have diagnosis codes for ERCP complications [4]. As a result, the first two sets of diagnosis codes for each complication were removed as a way to filter out patients who had them prior to ERCP [9,13]. This method has been established and published in the literature to identify secondary diagnoses after ERCP has been performed [9,13]. However, upon switching to ICD-10, there were only 305 patients diagnosed with paralytic ileus in 2016 and 340 patients in 2017, from 3955 to 6016 patients in the years prior using ICD-9 (Appendix - Table 6). This decrease in the number of patients can be caused by the improper translation between two versions of the diagnosis codes. Lastly, patients with paralytic ileus who were critically ill were likely in the intensive care units, but NIS does not have data on the level or duration of care provided (e.g., intensive care, step-down care, or medical-surgical). This would be difficult to code into the database since patients are likely to be transferred across multiple units within the hospital admission. Unfortunately, these factors are inherent to the database and could not be accounted for during our analysis.

The strength of our study comes from the generalizability of the NIS inpatient database. By combining data across multiple years, we were able to increase our sample size and perform more stringent case-control matching to give us higher-quality results. This is one of the largest publicly available databases to study paralytic ileus since this diagnosis is mostly seen in hospitalized patients.

Our results raise questions for future studies on ERCP complications. Glucagon, which decreases duodenal motility, has been used to help with the cannulation of the sphincter of Oddi [30]. It will be worthwhile to analyze the rates of glucagon usage and their effects on outcomes for patients with paralytic ileus. Another idea to consider is the evaluation of the use of prokinetic agents, a potential treatment for paralytic ileus, to determine if restoring the functionality of the intestines can have a protective effect [1]. These studies can possibly help endoscopists minimize the morbidity and mortality associated with ERCP in the critically ill population.

## Conclusions

Hospitalized patients with paralytic ileus had a longer length of stay, higher average total charges, and fewer patients with some form of payment. They also had increased risks of post-ERCP complications such as infection, pancreatitis, cholangitis, cholecystitis, perforation, and hemorrhage, as well as overall mortality. These are likely due to critical illness and systemic inflammation. Therefore, patients with paralytic ileus should be medically optimized before undergoing ERCP to minimize the risks of complications.

## Appendices

**Table 4:** ICD-9 and ICD-10 procedure codes used for endoscopic retrograde cholangiopancreatography in the National Inpatient Sample database

Procedure codes	ICD-9	ICD-10
ERCP	51.10, 51.11, 52.13	BF110ZZ, BF111ZZ, BF11YZZ, BF100ZZ, BF101ZZ, BF10YZZ, BF180ZZ, BF181ZZ, BF18YZZ
Endoscopic biopsy of pancreatic duct	52.14	0FBD8ZX
Endoscopic excision or destruction of lesion or tissue of pancreatic duct	52.21	0F5D8ZZ, 0FBD8ZZ
Endoscopic excision or destruction of lesion of biliary ducts or sphincter of Oddi	51.64	0F568ZZ, 0F558ZZ, 0F578ZZ, 0F588ZZ, 0F598ZZ, 0F5C8ZZ, 0FB58ZZ, 0FB68ZZ, 0FB78ZZ, 0FB88ZZ, 0FB98ZZ, 0FBC8ZZ, 0FB48ZX, 0FB58ZX, 0FB68ZX, 0FB78ZX, 0FB88ZX, 0FB98ZX, 0FBC8ZX
Cannulation of pancreatic duct, Endoscopic dilation of pancreatic duct	52.92, 52.98	0F7D7DZ, 0FHD7DZ, 0F7D8ZZ, 0F7D8DZ
Endoscopic insertion of stent into pancreatic duct	52.93	0FHD8DZ
Endoscopic removal of stone from pancreatic duct	52.94	0FCD8ZZ
Endoscopic insertion of nasopancreatic drainage tube	52.97	0F9D80Z
Endoscopic biopsy of biliary duct or sphincter of Oddi	51.14	0F958ZX, 0F968ZX, 0F978ZX, 0F988ZX,0F998ZX, 0F9C8ZX
Endoscopic removal of stone from biliary tract	51.88	0FC58ZZ, 0FC68ZZ, 0FC78ZZ, 0FC88ZZ, 0FC98ZZ, 0FCC8ZZ, 0FF48ZZ, 0FF58ZZ, 0FF68ZZ, 0FF78ZZ, 0FF88ZZ, 0FF98ZZ, 0FFC8ZZ
Endoscopic insertion of stent into bile duct	51.87	0F758DZ, 0F768DZ, 0F778DZ, 0F788DZ, 0F798DZ, 0FHB4DZ, 0FHB8DZ
Endoscopic insertion of nasobiliary drainage tube	51.86	0F9980Z
Endoscopic sphincterotomy and papillotomy	51.85	0F9C80Z
Endoscopic dilation of ampulla and biliary duct	51.84	0F758ZZ, 0F768ZZ, 0F778ZZ, 0F788ZZ, 0F798ZZ, 0F7C8DZ, 0F7C8ZZ, 0F968ZZ, 0F978ZZ, 0F988ZZ, 0F998ZZ, 0F9C8ZZ, 0F9D8ZZ
Exploration of common duct	51.51	0F9940Z

**Table 5:** ICD-9 and ICD-10 diagnosis codes for paralytic ileus and post-endoscopic retrograde cholangiopancreatography complications

Diagnosis codes	ICD-9	ICD-10
Paralytic ileus	560.1	K56.0
Post-ERCP infection (sepsis, severe sepsis, septic shock, bacteremia)	997.49 and (038.9, 995.91, 995.92, or 785.52)	K91.89 and (T81.4XXA, A41.9, R65.20, R65.21, or R78.81)
Post-ERCP pancreatitis	997.49 and (577.0)	K91.89 and (K85.90, K85.91, K85.92)
Post-ERCP cholangitis	997.49 and (576.1)	K91.89 and (K83.09)
Post-ERCP cholecystitis	997.49 and (575.0)	K91.89 and (K81.0)
Post-ERCP perforation	997.49 and (998.2 or 576.3)	K91.89 and (K91.71, K83.2)
Post-ERCP hemorrhage	997.49 and (998.11)	K91.89 and (K91.840)

**Table 6:** Number of endoscopic retrograde cholangiopancreatography procedures and cases of paralytic ileus from 2007 to 2017 in the National Inpatient Sample Database

Year	No. of ERCP procedures	No. of paralytic ileus cases
2007	161,843	4645
2008	177,955	5745
2009	176,156	6016
2010	181,818	6008
2011	183,845	5953
2012	173,325	5320
2013	172,305	5335
2014	173,780	5440
2015	181,530	3955
2016	211,560	305
2017	214,180	340
